# Anti-Inflammatory Effect of IL-37b in Children with Allergic Rhinitis

**DOI:** 10.1155/2014/746846

**Published:** 2014-08-11

**Authors:** Wenlong Liu, Li Deng, Yanqiu Chen, Changzhi Sun, Jie Wang, Lifeng Zhou, Huabin Li, Renzhong Luo

**Affiliations:** ^1^Department of Otolaryngology, Guangzhou Women and Children's Medical Center, Guangzhou Medical College, No. 9 Jinsui Road, Guangzhou 510623, China; ^2^Department of Otolaryngology, Head and Neck Surgery, Xinhua Hospital, Shanghai Jiaotong University School of Medicine, No. 1665 Kongjiang Road, Shanghai 200092, China

## Abstract

*Background*. Interleukin-37 (IL-37), a newly described member of IL-1family, functioned as a fundamental inhibitor of innate inflammatory and immune responses, especially its isoform IL-37b. *Objective*. This study was undertaken to evaluate the expression and regulation of IL-37b in children with allergic rhinitis (AR). *Methods*. Forty children with AR and twenty-five normal controls were included. The relationship between IL-37b and Th1/2 cytokines production in serum and nasal lavage was examined by enzyme-linked immunosorbent assay (ELISA). Peripheral blood mononuclear cells (PBMCs) were purified for in vitro regulation experiment of IL-37b. Intranasal mometasone furoate was given in AR children and IL-37b change after one-month treatment was detected using ELISA. *Results*. We observed significantly decreased IL-37b expression levels in both serum and nasal lavage compared to controls. IL-37b was negatively correlated with Th2 cytokines. Our results also showed that IL-37b downregulated Th2 cytokine expressed by PBMCs and this modulation was through mitogen-activated protein kinase (MAPK) and phosphatidylinositol 3-kinase (PI3K) pathway. We also found that intranasal mometasone furoate therapy can promote nasal IL-37b expression. *Conclusion*. IL-37b may be involved in Th2 cytokine regulation in AR and its expression was related to the efficacy of intranasal steroid therapy.

## 1. Introduction 

Allergic rhinitis (AR) is a worldwide common disease in children with occurrence of 10–40% [1, 2]. It affects both life and performance of children and it is often accompanied by asthma [3]. T cell type-2 (Th2) inflammation accompanied by infiltration with eosinophils is characteristic pathological changes in AR [4, 5]. Infiltration of T cells, B cells, plasma cells, eosinophils, basophils, and neutrophils and activation of Th2 cells caused the occurrence of AR [6]. Most studies about pathophysiological mechanism of AR focused on proinflammatory cytokines, while anti-inflammatory cytokines received less attention.

Interleukin-37 (IL-37) is a recently discovered anti-inflammatory cytokine and can bind to IL-18-receptor (R) and IL-18-binding protein (BP) [7, 8]. IL-37 can be secreted by monocytes, macrophages, epithelial cells, and so on [9]. IL-37 is expressed in normal and tumor tissues in human and it exerts anti-inflammatory effects by suppressing innate immune responses through decreasing the production of inflammatory cytokines [10]. IL-37 has five different isoforms, of which IL-37b is the largest isoform and it is believed to suppress proinflammatory cytokine production and dendritic cell (DC) activation [8]. The role of IL-37b in autoimmune diseases was well characterized recently. It was reported that IL-37b expression was elevated in rheumatoid arthritis, in patients with* Mycobacterium avium* infections, atherosclerotic coronary and carotid artery plaques, in psoriatic plaques, and in Crohn's disease [10, 11]. However, the role of IL-37b in allergic disease is not characterized.

In our present study, we sought to determine the expression of IL-37b in serum and nasal lavage of AR children and the effect of IL-37b on Th1/2 cytokines production and the involved signaling pathway.

## 2. Methods

### 2.1. Patients

Forty children (<18 years old) with AR were recruited in the study. The diagnosis was established on history, clinical examination, skin prick test, and specific IgE measurement, which is in accordance with Allergic Rhinitis and its Impact on Asthma (ARIA) guideline (2010) [12]. Of them, children with asthma were diagnosed according to the global initiative for asthma (GINA) criteria [13]. Twenty-five children of similar age and gender with no history of allergic disease or wheezing were selected as the control group. Those with chronic diseases (e.g., malnutrition, anatomic malformation of the respiratory system, chronic lung disease, heart disease, gastroesophageal reflux disease, and cystic fibrosis) and those with a history of chronic drug use (e.g., oral or nasal corticosteroids, antiepileptics, and immune suppressives) were excluded from the study. The tissues from the inferior turbinate from nine AR children (13–17 years old) and eight controls (13–16 years old) that underwent inferior turbinectomy due to severe nasal congestion caused by inferior turbinate hypertrophy were sampled for detection.

### 2.2. Blood and Nasal Lavage Samples Preparation and Analysis

Venous blood samples were collected into Vacuette tubes and centrifuged at 1000 g for 15 min at 4°C. Serum samples were stored at −80°C. These samples were used for enzyme-linked immunosorbent assay (ELISA) and qPCR measurement. The whole blood cell counts were measured by LH-785 system (Beckman Coulter, Mervue, Galway, Ireland). Total serum IgE was measured by electrochemiluminescence (ECLIA) method using an ELX-800 system.

Nasal lavage was performed using saline warmed to 37°C. The process was performed according to method described elsewhere [14]. Due to cooperation of children, 28 AR samples and 15 controls were collected. The samples were centrifuged to remove cellular debris and aliquots of the supernatants were stored at −20°C in eppendorf tubes until analysis. Total protein concentrations were determined with Bio-Rad protein assays according to Bradford [15].

### 2.3. ELISA for Protein Expression and ECLIA for Eosinophil Cationic Protein (ECP) Protein Expression

ELISA kits were used for measuring IL-37b, IL-4, IL-13, IL-5, IL-12, IFN-*γ*, and IL-10 (all from R&D systems, USA) level in serum, nasal lavage, and supernatant of peripheral blood mononuclear cells (PBMCs). ECP levels were detected by other ELISA kit (EIAab, Wuhan, China), according to the manufacturer's protocols. The detection limits of the assays were as follows: IL-37b (125 pg/mL), IL-4 (1.56 pg/mL), IL-13 (93.8 pg/mL), IL-5 (7.8 pg/mL), IL-12 (2.5 pg/mL), IFN-*γ* (12.5 pg/mL), and IL-10 (3.9 pg/mL).

### 2.4. Real-Time PCR Analysis

Real-time PCR was performed as described previously [16]. Total RNA was extracted from mucosa tissues using TRIzol reagent (Life Technologies, Carlsbad, CA, USA) according to the manufacturer's instructions. Reverse transcription (RT) was performed, and cDNA was synthesized from 2 *μ*g of total RNA using an oligo (dT) 18 primer and M-MLV reverse transcriptase (TAKARA, Syuzou, Shiga, Japan). The mRNA expression was determined using an ABI PRISM 7300 Detection System (Applied Biosystems, Foster City, CA, USA) and SYBR Premix Taq (TAKARA). The sequences of the primers were as follows: IL-37b, forward primer: 5′-CTCCTGGGGGTCTCTAAAGG-3′, reverse primer: 5′-TACAATTGCAGGAGGTGCAG-3′; *β*-actin, forward primer: 5′-CAGAGCAAGAGAGGCATCCT-3′, reverse primer: 5′-GTTGAAGGTCTCAAACATGATC-3′; PRISM samples contained 1× SYBR Green Master Mix, 1.5 *μ*L of 5 *μ*M primers, and 25 ng of synthesized cDNA in a 25 *μ*L volume. Reaction mixtures were heated to 95°C for 10 min, followed by 40 cycles of denaturation at 95°C for 10 s, and annealing extension at 60°C for 60 s. All PCR reactions were performed in duplicate. Melting curve analysis was used to control for amplification specificity. The mean value of the replicates for each sample was calculated and expressed as a cycle threshold (Ct) value. The relative expression of each target gene was determined as the difference (ΔCt) between the Ct value of the target gene and the Ct value of *β*-actin. Fold changes in the target gene mRNA were determined as 2^−ΔΔCt^.

### 2.5. Immunohistochemical (IHC) Staining

For immunohistochemistry, the slides were placed in 0.3% H_2_O_2_ for 20 minutes at room temperature to reduce nonspecific background staining caused by endogenous peroxidases. After additional washing with PBS again, the slides were boiled in 10 mM citrate buffer for 15 minutes followed by cooling at room temperature. Antibodies for IL-37b (Abcam and Creative BioMart, USA), IL-12 (R&D systems, USA), IL-4 (R&D systems, USA), IL-1 (R&D systems, USA), and IgG (as control, Dako, Denmark) were incubated overnight at 4°C for immunohistochemical staining, respectively. The next day, the slides were washed with PBS and incubated with secondary antibody conjugated with streptavidin-horseradish peroxidase (Gene Tech, Shanghai, China) at room temperature for 1 hour.

After washing, DAB (Gene Tech, Shanghai, China) staining was performed under microscope. After rinsing with distilled water, the sections were counterstained with Mayer's hematoxylin (Zhongshan Goldenbridge, Beijing, China) for further 25 seconds, dehydrated with series of ethanol (v/v, 90%–100%), cleared from lipid with xylene (3 times), and mounted with neutral balsam (Zhongshan Goldenbridge, Beijing, China). Control for nonspecific staining was routinely performed with PBS but antibody and all proved negative with secondary antibody.

The sections were blindly examined and coded with no awareness of the clinical data. They were visualized with an Olympus CX40 Microscope (Olympus Europa GmbH, Germany). The number of positive cells (brown cells) was counted in 10 high-magnification visual fields (×200) and averaged.

### 2.6. PBMCs Preparation

PBMCs were separated from 20 mL of heparinized whole blood from AR children. Specifically, cells were isolated by Lymphoprep (Fresenius Kabi Norge AS, Oslo, Norway) density-gradient centrifugation from heparinized leucocyte-enriched buffy coats. Then, PBMCs were cultured at 2∗10^6^/mL in 24-well plates in culture medium: RPMI 1640 supplemented with 5% human AB serum, 5 mmol/L glutamine, and penicillin, and streptomycin solution (all from Invitrogen, except for serum from Sigma-Aldrich). Stimulation was performed through addition of rhIL-37b (1–100 ng/mL) with or without other cytokines (Poly (I:C) (1 ng/mL), rhIL-4 (1–100 ng/mL), rhIL-5 (1–100 ng/mL), rhIL-13 (1–100 ng/mL), rhIL-12 (1–100 ng/mL), rhIFN-*γ* (1–100 ng/mL), rhIL-10 (1–100 ng/mL), SB203580 (10 *μ*mol/L), and LY294002 (10 *μ*mol/L). All the above stimulators were from R&D systems.

### 2.7. Human Peripheral Blood Eosinophil Purification and Assay

Eosinophils were obtained from the peripheral blood of atopic donors (blood eosinophil levels 5–10%) by MACS-negative immunomagnetic separation as described previously [17]. The purity of cells was 98–100% (Kimura staining) and the viability was larger than 98% (Trypan blue staining). The isolated eosinophils were suspended in RPMI-1640 supplemented with 10% heat-inactivated fetal calf serum (FCS), 2 mM L-glutamine, 100 U/mL penicillin, and 100 *μ*g/mL streptomycin. Eosinophils (500 *μ*L, 10^5^/mL) were treated with or without rh-IL-37 (1–100 ng/mL) and eotaxin (1 ng/mL). ECP protein changes were detected by Unicap system.

### 2.8. Intranasal Mometasone Furoate Therapy

All AR children underwent a 4-week course of intranasal mometasone furoate (50 *μ*g per puff per nostril to both nostrils, once daily). At the end of treatment, the nose symptoms (runny nose, sneezing, and blocked nose), eye symptoms (streaming and swelling, redness and itching), and lung symptoms (breathlessness, cough, wheeze and chest tightness) were scored by the children as follows: 0 = no symptoms, 1 = slight symptoms, 2 = moderate symptoms, and 3 = severe symptoms. Besides, blood and nasal lavage samples were collected and changes of IL-37b protein expression were detected with ELSIA.

### 2.9. Statistical Analysis

All data were expressed as mean ± SD except additional note. Statistical significance between different groups was determined using nonparametric Mann-Whitney *U* test. The Spearman rank correlation test was used to analyze the correlation among the expression of biomarkers and clinical stage. *P* < 0.05 was considered as significant difference.

## 3. Results

### 3.1. Demographic and Laboratory Characteristics of the Study Population

This study was conducted with 65 children, 40 of whom suffered from AR, with ages ranging between 16 and 187 months (mean age: 73.2 ± 33.0 months, 22 males), and 25 of whom were healthy controls with ages ranging between 15 and 184 months (mean age: 72.0 ± 31.8 months, 11 males). The demographic features and laboratory parameters of the population are presented in Table 1. Asthma was found to be 25% in the AR children. When the two groups were compared, the basophil count, the eosinophil count, total serum IgE, and ECP levels were found to be higher in the AR without asthma group than those of the control group, especially in AR with asthma group.

### 3.2. Decreased IL-37b mRNA and Protein Levels in Relation to Th1/Th2/Treg Cytokines in AR

**Figure 1:**
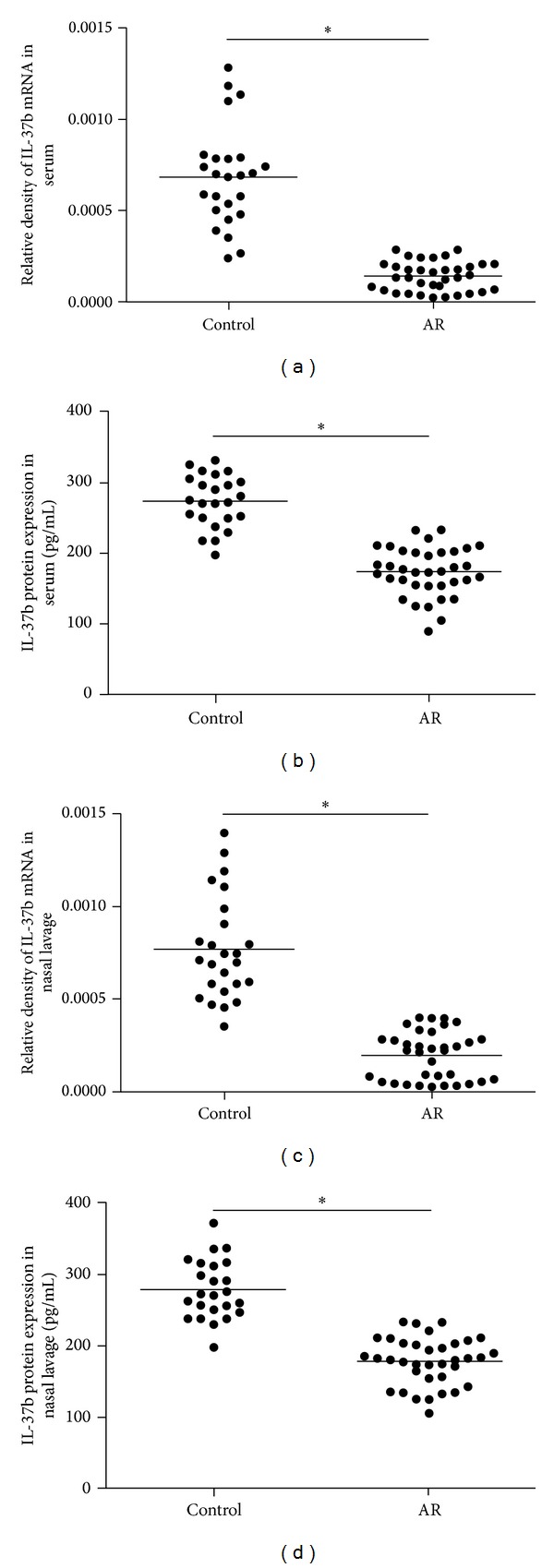
The serum and nasal lavage mRNA and protein expression of IL-37b in AR and normal control. (a) The serum IL-37b mRNA level in AR and normal control; (b) the serum IL-37b protein level in AR and normal control; (c) the nasal lavage IL-37b mRNA level in AR and normal control; (d) the nasal lavage IL-37b protein level in AR and normal control. *P < 0.05, comparing the two groups.

**Figure 2:**
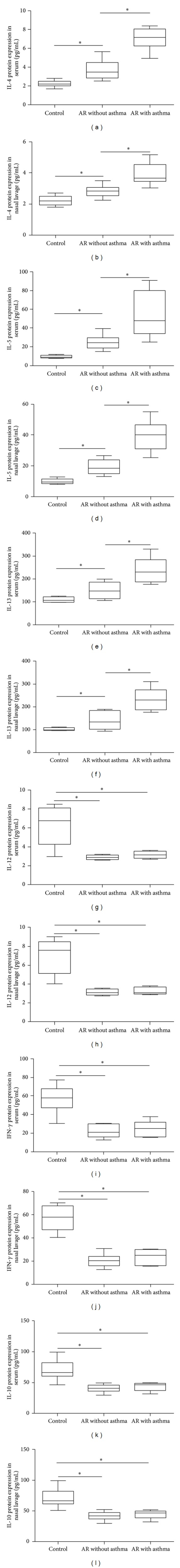
The serum and nasal lavage protein expression of Th2 (IL-4, IL-5, and IL-13), Th1 (IL-12, IFN-*γ*) and Treg (IL-10) cytokines in AR with asthma, AR without asthma, and control groups. (a)–(f) The serum and nasal lavage protein expression of Th2 (IL-4, IL-5, and IL-13) cytokines in AR with asthma, AR without asthma, and control groups; (g)–(j) the serum and nasal lavage protein expression of Th1 (IL-12, IFN-*γ*) cytokines in AR with asthma, AR without asthma, and control groups; (k), (l) the serum and nasal lavage protein expression of Treg (IL-10) cytokines in AR with asthma, AR without asthma, and control groups. *P < 0.05, comparing the two groups.

**Figure 3:**
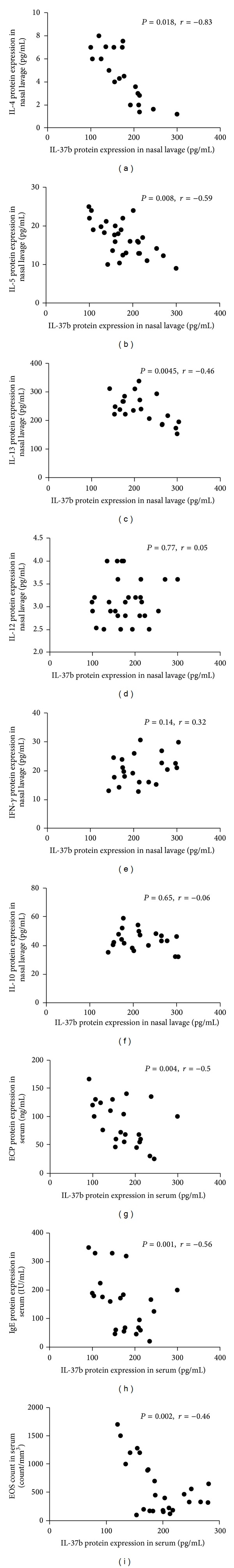
Correlation between IL-37b protein expression and related cytokines or protein. (a) Correlation between nasal IL-37b and IL-4 protein; (b) correlation between nasal IL-37b and IL-5 protein; (c) correlation between nasal IL-37b and IL-13 protein; (d) correlation between nasal IL-37b and IL-12 protein; (e) correlation between nasal IL-37b and IFN-*γ* protein; (f) correlation between nasal IL-37b and IL-10 protein; (g) correlation between serum IL-37b and ECP protein; (h) correlation between serum IL-37b and IgE protein; (i) correlation between serum IL-37b and EOS count.

The serum and local IL-37b mRNA and protein expression in AR were significantly lower than those in the normal controls (*P* < 0.001, Figures 1(a)–1(d)). However, when we subdivided the AR group into asthma and nonasthma group, we found no difference of IL-37b expression levels between two groups. We also compared IL-37 expression between AR children >6 yr and <6 yr and no differences were found (data not shown). Our results showed enhanced IL-4, IL-13, and IL-5 protein expression, especially in children with asthma and decreased IL-12, IFN-*γ*, and IL-10 protein expression in serum and nasal lavage of AR children compared with control group (*P* < 0.001, Figures 2(a)–2(l)). Nasal IL-4, IL-5, and IL-13 were negatively correlated with local IL-37b (*P* < 0.05, Figures 3(a)–3(c)). The serum ECP and IgE levels as well as eosinophil counts were found to be negatively correlated with serum IL-37b expression (*P* < 0.05, Figures 3(g)–3(i)). No relationship between IL-37b and IL-12, IFN-*γ*, and IL-10 was found (Figures 3(d)–3(f)).

### 3.3. Decreased IL-37b Expression in AR by IHC

**Figure 4:**
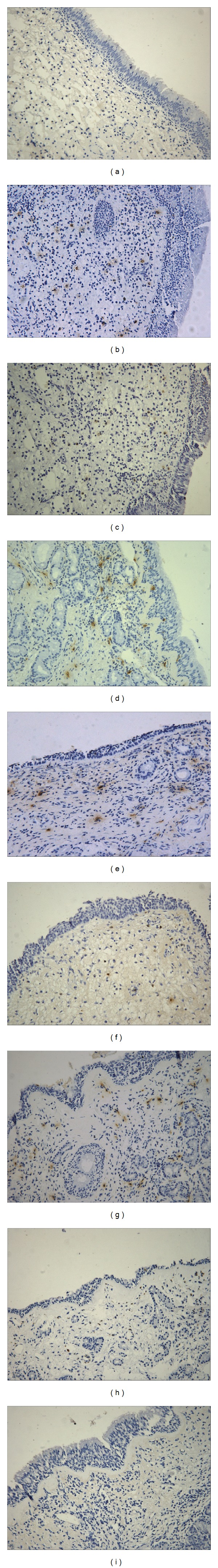
The expression of IL-37b and related cytokines in inferior turbinate of AR children and normal control. (a) IL-37b staining in AR; (b) IL-37b staining in control; (c) IL-12 staining in AR; (d) IL-12 staining in control; (e) IL-4 staining in AR; (f) IL-4 staining in control; (g) IL-1 staining in AR; (h) IL-1 staining in control; (i) IgG control antibody staining. Magnification ×200.

In normal tissues, some epithelial cells, interstitial cells, and glandular cells were positive for IL-37b and IL-37b immunoreactivity was enhanced significantly in controls compared with AR samples (Figures 4(a) and 4(b)). We also detected IL-12 (Figures 4(c) and 4(d)), IL-4 (Figures 4(e) and 4(f)), and IL-1 (Figures 4(g) and 4(h)) and the results showed that the number of IL-4 and IL-1 positive cells in AR was significantly higher than that of control, while the IL-12 positive cells presented as opposite trend (Table 2).

### 3.4. IL-37b Regulated Th2 Cytokines Expression in PBMCs through Mitogen-Activated Protein Kinase (MAPK) and Phosphatidylinositol3-Kinase (PI3K) Pathway

**Figure 5:**
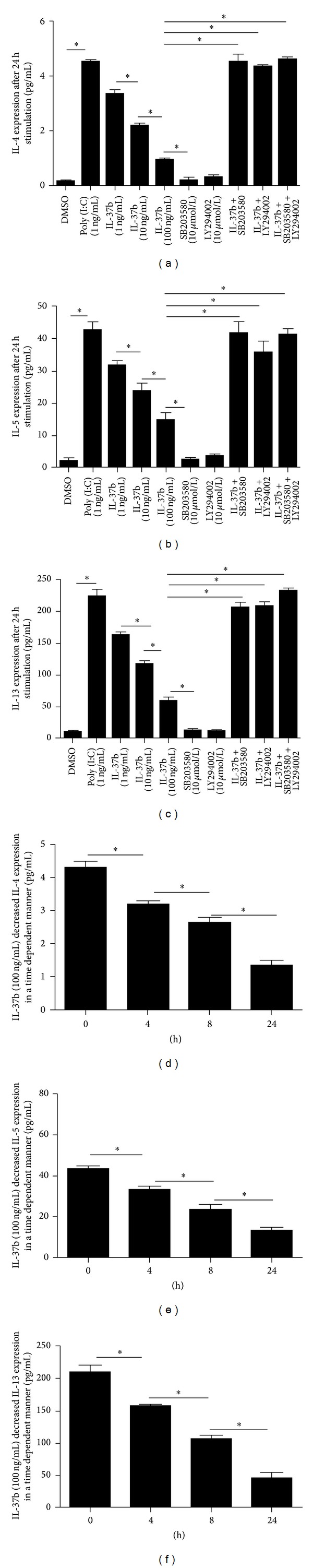
The regulation of Th2 cytokines (IL-4, IL-5, and IL-13) by IL-37b in PBMC in a dose and time dependent manner. (a) IL-4 expression by PBMC after stimulated with different concentration of IL-37b with or without SB203580 and LY294002; (b) IL-5 expression by PBMC after stimulated with different concentration of IL-37b with or without SB203580 and LY294002; (c) IL-13 expression by PBMC after stimulated with different concentration of IL-37b with or without SB203580 and LY294002; (d) IL-37b decreased IL-4 expression by PBMC in a time dependent manner; (e) IL-37b decreased IL-5 expression by PBMC in a time dependent manner; (f) IL-37b decreased IL-13 expression by PBMC in a time dependent manner. *P < 0.05, comparing the two groups.

**Figure 6:**
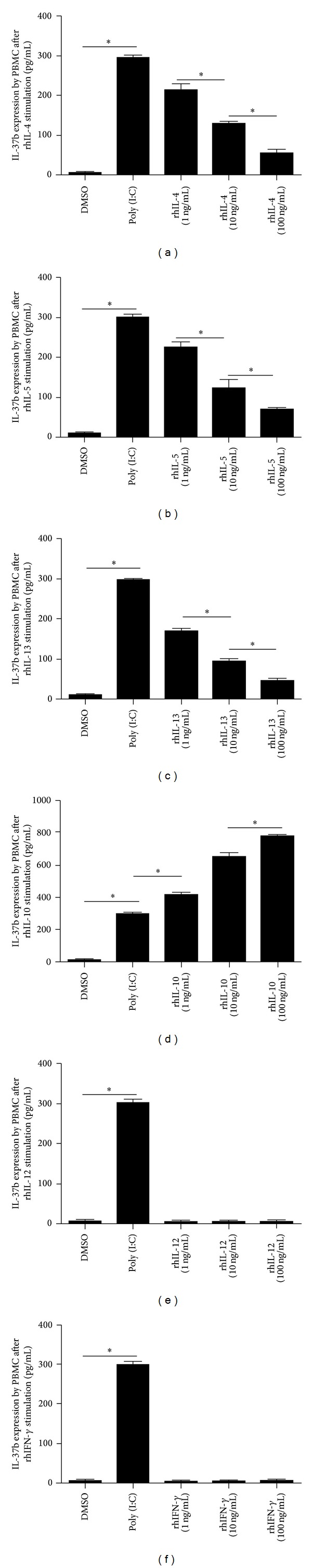
Regulation of IL-37b expression by Th2 cytokines (rhIL-4, rhIL-5, rhIL-13), Th1 cytokines (rhIL-12, rhIFN-*γ*), and Treg cytokine (rhIL-10) in PBMC. (a) IL-37b expression in a dose dependent manner by PBMC after 24 h stimulation with rhIL-4; (b) IL-37b expression in a dose dependent manner by PBMC after 24 h stimulation with rhIL-5; (c) IL-37b expression in a dose dependent manner by PBMC after 24 h stimulation with rhIL-13; (d) IL-37b expression in a dose dependent manner by PBMC after 24 h stimulation with rhIL-10; (e) IL-37b expression was not affected after 24 h stimulation with rhIL-12; (f) IL-37b expression was not affected after 24 h stimulation with rhIFN-*γ*. *P < 0.05, comparing the two groups.

After stimulation with various concentrations of rhIL-37b (1–100 *μ*g), we found significant downregulation of Th2 cytokines (IL-4, IL-5, and IL-13) in a dose and time dependent manner and this effect was blocked after treatment with SB203580 and LY294002 (Figures 5(a)–5(f)). However, our results showed that IL-37b did not regulate Th1 cytokines and IL-10 expression. To investigate the effect of Th1/Th2/Treg cytokines on IL-37b expression, we used rhIL-4, rhIL-13, rhIL-5, rhIL-12, rhIFN-*γ*, and rhIL-10 to stimulate PBMCs for 24 hours and found that Th2 cytokines (rhIL-4, rhIL-13, and rhIL-5) decreased IL-37b expression (Figures 6(a)–6(c)), whereas Th1 cytokines (rhIL-12, rhIFN-*γ*) had no effect on IL-37b expression (Figures 6(e) and 6(f)). Interestingly, rhIL-10 can upregulate IL-37b expression by PBMCs (Figure 6(d)).

### 3.5. IL-37b Induces ECP Production by Eosinophils

**Figure 7:**
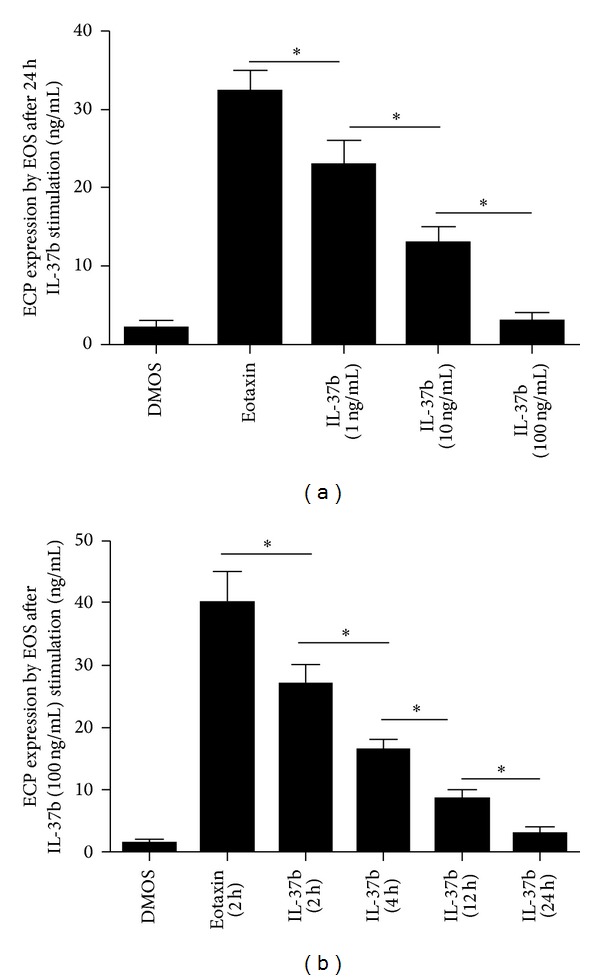
Regulation of ECP expression by EOS after stimulation with IL-37b. (a) ECP expression in a dose dependent manner by EOS after stimulation with rhIL-37b; (b) ECP expression in a time dependent manner by EOS after stimulation with rhIL-37b. *P < 0.05, comparing the two groups.

After stimulation with various concentrations of rhIL-37b (1–100 ng/mL), we found significant downregulation of ECP level by eosinophils in a dose and time dependent manner (Figures 7(a) and 7(b)).

### 3.6. Intranasal Mometasone Furoate Therapy Downregulates Local IL-37b Expression

**Figure 8:**
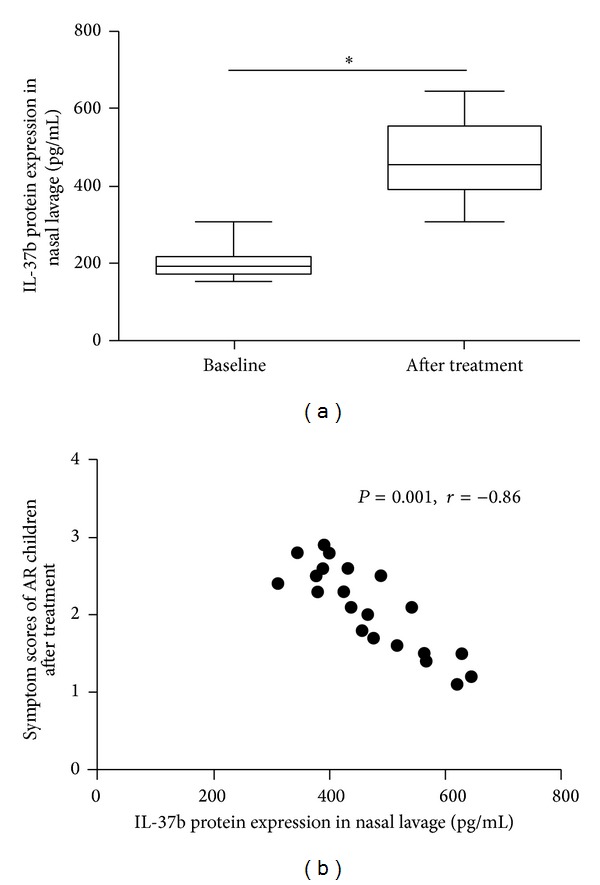
Nasal lavage IL-37b protein expression after treatment and its correlation with total symptom scores. (a) Nasal lavage IL-37b protein expression before and after treatment. (b) Correlation between nasal lavage IL-37b protein expression and total symptom scores after treatment. *P < 0.05, comparing the two groups.

After 4-week course of intranasal mometasone furoate (50 *μ*g per puff per nostril to both nostrils, once daily), we found significant relieve of symptoms of AR children (Table 3). Nasal lavage IL-37b protein expression was enhanced significantly after treatment and its expression was negatively related with total symptom scores (Figures 8(a) and 8(b)).

## 4. Discussion

AR is a common disease in children which is characterized by persistent inflammation of the nasal mucosa, typically showing a Th2 skewed eosinophilic inflammation with high levels of IL-5 and IgE. Th2 cytokines play an important role in the development and deterioration of AR. At present, intranasal steroid is the first-line treatment for AR and its mechanism includes regulation of Th1/Th2 balance and inhibition of inflammatory cytokines.

At present, most studies on pathogenesis of AR concentrated on the production of inflammatory cytokines instead of anti-inflammatory cytokines. IL-37 (IL-1F7) is a newly reported molecular of IL-1 family with anti-inflammatory effect [18]. IL-37 expression was found in many cancer cells such as stroma of colon carcinomas, and ductal mammary carcinoma and also in blood monocytes, fully differentiated keratinocytes in stratum granulosum of skin, PBMC, and dendritic cell [19, 20]. IL-37b has potent anti-inflammatory properties and many studies have elucidated its precise role in autoimmune disease such as systemic lupus erythematosus (SLE), rheumatoid arthritis (RA), inflammatory bowel disease (IBD), guillain-Barré syndrome, and atopic dermatitis (AP) [21, 22]. However, its role in respiratory disease was not reported.

As IL-37b was believed to be the most important isoform of IL-37, thus our study focuses on it and we provide the first evidence that IL-37b expression was negatively related to enhanced Th2 inflammation in AR. In normal nasal mucosa, we found that IL-37b was expressed by epithelial, interstitial cells, and glandular cells. However, IL-37b expression in AR was relatively weak. Interestingly, we found that IL-37b expression was not affected by the state of asthma and age in children. Consistent with our results, Fujita et al. [22] detected IL-37b expression in AP and their results showed that most AP patients presented as high IL-37 level, but several severe AD showed low level of IL-37. These results suggested that IL-37 played different roles in different disease states and IL-37 isoforms may represent even different functions. Besides, when MAPK and PI3K pathway inhibitor were added to PBMCs, the inhibitory effect on Th2 cytokines by IL-37b was significantly decreased, suggesting that MAPK and PI3K pathways were involved in IL-37b mediated Th2 inflammation regulation, which was consistent with previous report [23].

In Imaeda's study [23], IL-37b was found to inhibit CXCL10, a Th1-chemokine, suggesting its potential role in inhibiting Th1 inflammation. On the contrary, our data showed that Th1 cytokines were not correlated with IL-37b. We also found that IL-37b did not affect IL-10 expression. These results suggested that IL-37 regulation of Th2 cytokines is not IL-10 dependent, which is inconsistent with Mcnamee's study [11], who reported that IL-37 isoforms not only suppress Th2 cytokines, but also induce IL-10. However, our data showed that rhIL-10 can upregulate IL-37b expression by PBMCs and its mechanism needs further exploration.

Eosinophils mediate epithelial damage via the release of preformed effector molecules, such as major basic protein and ECP. Eosinophils play a critical role in the maintenance and progression of AR by promoting airway dysfunction and tissue remodeling. Our results show serum ECP and IgE levels and eosinophil counts were negatively correlated with serum IL-37b. Thus, we purified eosinophils and found that IL-37b can inhibit ECP expression by eosinophils directly in a dose and time dependent manner. Our results provide a new eosinophil regulator in the development of AR.

Finally, we analyzed the IL-37b expression after intranasal steroid treatment. We found the treatment decreased nasal IL-37b expression and disease symptoms, suggesting that intranasal steroid may improve symptoms through regulation of IL-37b expression. However, inconsistent with our results, Song's study [24] showed that glucocorticoid can downregulate the expression of IL-37b and other cytokines in SLE patients. These differences in expression of IL-37b in above-mentioned diseases suggested that IL-37b played various roles in different backgrounds.

## 5. Conclusions

In summary, we have shown the expression, distribution, and regulation of IL-37b in AR children and demonstrated the importance of decreased IL-37b level in regulation of Th1/Th2/Treg balance and activation of eosinophils. Our findings may be beneficial for designing a potential strategy for the optimal prevention and management of AR children.

## Figures and Tables

**Table 1:** Demographic characteristic of AR children and normal controls.

Groups	AR without asthma group	AR with asthma group	Control
Number	30	10	25
Sex (male : female)	16 : 14	6 : 4	12 : 13
Age (months)	71.2 ± 34.0	63.2 ± 23.0	72.0 ± 31.8
History of asthma, *n* (%)	0	100	0
Family history of atopy, *n* (%)	15 (50)∗	8 (80)^#^	3 (12)
Exposure to smoking, *n* (%)	18 (60)∗	7 (70)^#^	1 (4)
Eosinophil^a^ (count/mm^3^)	145 (60–1350)∗	195 (60–1350)^#^	73 (5–230)
Neutrophil^a^ (count/mm^3^)	8430 (2000–16300)	12330 (9000–25700)^#^	7400 (3200–12500)
Basophil^a^ (count/mm^3^)	50 (10–150)∗	160 (70–350)^#^	8 (4–16)
Monocytes^a^ (count/mm^3^)	238 (70–2740)	268 (80–1960)	256 (60–2530)
Lymphocyte^a^ (count/mm^3^)	7900 (3100–15400)	7500 (4100–16600)	8300 (2500–16300)
ECP^a^ (ng/mL)	38.2 (4.0–131.0)∗	68.1 (35.0–188.0)^#^	10.9 (3.6–109.0)
IgE^a^ (IU/mL)	101.1 (2.5–1200.0)∗	198.2 (87.5–1600.0)^#^	31.0 (5.3–89.0)

^a^Data presented as median values (minimum–maximum).

∗Compared with control group, *P* < 0.05.

^#^Compared with AR group, *P* < 0.05.

**Table 2:** Cell count of IL-37b and related cytokines in AR and control tissue (median ± IQR).

Groups	AR group	Control group
IL-37b	4.8 ± 2.3∗	16.4 ± 4.3
IL-4 IL-12	12.3 ± 4.5∗ 11.3 ± 5.6∗	4.5 ± 1.9 22.1 ± 7.8
IL-1	25.2 ± 8.1∗	8.1 ± 2.4

∗Compared with control group, *P* < 0.05.

**Table 3:** Clinical outcome of 40 AR children after treatment (median ± IQR).

	Before treatment	After treatment
Symptom scores		
nose symptom	2.3 ± 0.5	0.7 ± 0.1∗
lung symptom	1.5 ± 0.4	1.0 ± 0.2∗
eye symptom	1.8 ± 0.3	0.9 ± 0.4∗

Total scores	5.6 ± 1.2	2.4 ± 0.5∗

∗Compared with baseline level, *P* < 0.05.
